# Whole-genome sequencing and transcriptome-characterized *in vitro* evolution of aminoglycoside resistance in *Mycobacterium tuberculosis*

**DOI:** 10.1099/mgen.0.001022

**Published:** 2023-05-24

**Authors:** Wenjing Wei, Yuchuan Zhao, Chenchen Zhang, Meiling Yu, Zhuhua Wu, Liuyue Xu, Kehao Peng, Zhilong Wu, Yanxia Li, Xuezhi Wang

**Affiliations:** ^1^​ Center for Tuberculosis Control of Guangdong Province, Key Laboratory of Translational Medicine of Guangdong, Guangzhou 510630, PR China; ^2^​ Foshan Fourth People's Hospital, Foshan 528000, PR China

**Keywords:** *Mycobacterium tuberculosis*, aminoglycoside resistance, whole genome sequencing, transcriptome sequencing

## Abstract

Antibiotic resistance of *Mycobacterium tuberculosis* (Mtb) is a major public health concern worldwide. Therefore, it is of great significance to characterize the mutational pathways by which susceptible Mtb evolves into drug resistance. In this study, we used laboratory evolution to explore the mutational pathways of aminoglycoside resistance. The level of resistance in amikacin inducing Mtb was also associated with changes in susceptibility to other anti-tuberculosis drugs such as isoniazid, levofloxacin and capreomycin. Whole-genome sequencing (WGS) revealed that the induced resistant Mtb strains had accumulated diverse mutations. We found that *rrs* A1401G was the predominant mutation in aminoglycoside-resistant clinical Mtb isolates from Guangdong. In addition, this study provided global insight into the characteristics of the transcriptome in four representative induced strains and revealed that *rrs* mutated and unmutated aminoglycoside-resistant Mtb strains have different transcriptional profiles. WGS analysis and transcriptional profiling of Mtb strains during evolution revealed that Mtb strains harbouring *rrs* A1401G have an evolutionary advantage over other drug-resistant strains under the pressure of aminoglycosides because of their ultra-high resistance level and low physiological impact on the strain. The results of this study should advance our understanding of aminoglycoside resistance mechanisms.

## Data Summary

The sequence data have been deposited in the Sequence Read Archive (SRA) database at NCBI (https://www.ncbi.nlm.nih.gov/) under accession numbers PRJNA866200 and PRJNA870159 for DNA sequences, and PRJNA853078 for RNA sequences.

**Impact Statement**

Aminoglycoside resistance of *Mycobacterium tuberculosis* (Mtb) is a major public health concern worldwide. Although amikacin is classified as one of the class C adjunct drugs recommended by the World Health Organization for the long-term treatment of multidrug-resistant tuberculosis (MDR-TB), aminoglycosides still play an important role currently where MDR-TB prevention and second-line anti-TB drugs are limited, especially in developing countries. In this study, we used laboratory evolution of Mtb under increasing concentrations of aminoglycosides to explore the mutational pathways of aminoglycoside resistance. Whole-genome sequencing analysis and transcriptional profiling of Mtb strains during evolution revealed that the *rrs* A1401G mutant has an evolutionary advantage over the *rrs* C1402T mutant and wild-type *rrs* under high-concentration aminoglycoside stress. To some extent, this study explains why *rrs* A1401G had a high frequency in clinical strains resistant to kanamycin or amikacin, whereas *eis*, *whiB7*, *tlyA* and other mutations rarely appeared. These results provide a better understanding of the mechanisms involved in the development of aminoglycoside resistance. In addition, the mutational profile of clinical Mtb isolates in Guangdong Province revealed that only a small fraction of the strains contained classical drug-resistant mutation sites, suggesting that further research is needed to understand the mechanisms of drug resistance in order to explain the differences between genotypic and phenotypic approaches, which are not limited to certain antibiotics and certain isolates.

## INTRODUCTION

Tuberculosis (TB) is one of the deadliest infectious diseases worldwide. Every day, more than 4100 people die, and nearly 28 000 fall ill, from TB globally [1]. Drug resistance in *Mycobacterium tuberculosis* (Mtb) is a worldwide concern. In particular, the increasing number of drug-resistant TB cases, including rifampicin-resistant TB (RR-TB), multidrug-resistant TB (MDR-TB) and extensively drug-resistant TB (XDR-TB), has greatly threatened global health [1]. Guangdong has one of the highest TB burden in China. Chen *et al.* reported that there were 92 851 active pulmonary TB cases in Guangdong from 2016 to 2020. Among them, 30 256 Mtb strains were successfully cultured, with a total drug resistance rate of 26.75 and MDR of 3.34 %. The resistance rate of Mtb to streptomycin (SM) and kanamycin (KM) were 14.59 % (4430/30362) and 1.63 % (494/30362), respectively [2].

The treatment of drug-resistant TB is still dominated by chemotherapy [3]. Aminoglycosides have strong antibacterial activity against both Mtb and Gram-negative bacilli. Because of their clear efficacy and low cost, aminoglycosides play an important role in the treatment of MDR-TB [4]. Previously, the World Health Organization (WHO) recommended that patients with MDR-TB should use aminoglycosides in their treatment regimen for at least 6 months [5]. Given that aminoglycosides are associated with a high incidence of ototoxicity in MDR-TB treatment and may cause permanent hearing damage in patients, they were assigned as optional drugs of the group C adjuncts recommended by the WHO for the long-term treatment of MDR-TB in 2019 [6]. However, considering the difficulty in obtaining linezolid and bedaquiline and the high drug resistance rate of quinolones in most developing countries, aminoglycosides remain important drugs for the treatment of drug-resistant TB in these countries.

Aminoglycosides act on bacterial ribosomes and inhibit protein synthesis, thereby inhibiting bacterial survival [7]. In addition, they can increase the permeability of bacterial cell membranes, which facilitates the entry of drugs into bacteria [8]. The resistance mechanism of aminoglycosides in Mtb is mainly related to gene mutations, including *rrs* (Rvnr01) [9], *eis* (Rv2416c) [10, 11] and *whiB7* [12]. The point mutations A1401G, C1402T and G1484T within *rrs* are responsible for high-level resistance to amikacin (AKM) and KM [13]. KM resistance is also caused by mutations G-10A, C-12T and C-14T within the promoter region of *eis* [14], as well as *whiB7* (Rv3197A) [15]. Efflux pumps such as *Rv1258c* [13] and *Rv0194* have also been reported to be related to AKM and/or KM resistance, such as *Rv1258c* [16] and *Rv0194* [15]. Note that almost all of these studies were based on genome or targeted gene sequencing of clinical strains. Clinical strains have a complex background with many mutation types, some of which are related to lineage [17]. For example, even the most common mutation, *rrs* A1401G, was present in 84 % of KM-resistant strains and only approximately 50 % of AMK- or capreomycin (CAP)-resistant clinical isolates from mainland China [18]. The remaining resistance genotypes cannot be explained on the basis of known target site mutations, but must be explained by other mechanisms. Some researchers have attempted to understand the virulence and resistance to anti-TB drugs by observing the gene expression of Mtb exposed to drugs [19, 20].

In this study, using an *in vitro* induction assay with increasing concentrations of AKM or KM, we have established an evolutionary model of aminoglycoside-resistant Mtb to reveal how Mtb evolves from susceptibility to resistance and from low-level resistance to high-level resistance. By analysing the mutations in every generation of induced Mtb strains, we observed that *rrs* A1401G mutations were directly associated with KM and AKM, which was also the predominant mutation in aminoglycoside-resistant Mtb in Guangdong. Using RNA-sequencing (RNA-seq), this study provided global insight into the characteristics of the transcriptome in four representative induced strains and revealed that *rrs* mutated and unmutated aminoglycoside-resistant Mtb strains have different transcriptional profiles. The results of this study should advance our understanding of aminoglycoside resistance mechanisms and may improve the clinical understanding and rational use of aminoglycosides.

## METHODS

### Bacterial strains and growth conditions

The strains used in this study are listed in Tables S1, S4, S5 and S6, available in the online version of this article, and were cultivated as previously described [20]. Both the clinical and standard strains were obtained from the Center for Tuberculosis Control of Guangdong Province. All isolates were tested using both MYCOTB and the Löwenstein–Jensen (LJ) proportion method against 14 anti-TB drugs. Mtb strains were cultured on LJ medium, Middlebrook 7H9 and 7H10 media with OADC (oleic acid, albumin, dextrose and catalase) at 37 °C.

### *In vitro* induction of aminoglycoside-resistant Mtb

A monoclone of the Mtb H37Rv strain was selected as the primary strain (P0) to induce aminoglycoside resistance in the laboratory. Briefly, P0 was cultivated for approximately 4 weeks on LJ medium containing a continuous 2-fold gradient of the WHO recommended critical concentration (CC, 30 mg l^−1^ for AKM and KM), namely 2^−4^, 2^−3^, 2^−2^, 2^−1^ and 2^0^-fold of CC [20]. A drug-free culture was used as a control. Strains growing well were collected as the P1 strain. These steps were repeated until the minimal inhibitory concentration (MIC; defined as the lowest concentration of compound required to inhibit 99 % of bacterial growth over 3 weeks of incubation at 37 °C) of strains reached the CC (30 mg l^−1^), and the colony morphology was similar to that of the drug-free control group (P7). To stabilize the drug-resistant phenotype, P7 Mtb was continuously cultured on LJ medium containing 2^0^-fold of CC until the 10th generation (P10) for further analysis. Next, to obtain strains with high levels of aminoglycoside resistance, we cultured the P10 strains on LJ medium with 2-, 4- and 8-fold critical resistance concentrations of AKM or KM until the 27th generation, which grew well on 8-fold CC medium. The MICs and cross-drug resistance of all the lab-induced strains were rechecked using the Sensititre MYCOTB MIC plate (BASO), according to the manufacturer’s instructions. Each generation of strains produced during induction was stored in glycerol stocks at −80℃ for further analysis. For P3 Mtb induced by AKM and P4 Mtb induced by KM, 50 single colonies of each strain were selected and assessed by targeted sequencing of PCR amplicons of *rrs* associated with aminoglycoside resistance. Primers used are listed in Table S11.

### Whole genome resequencing

Mtb genomic DNA was extracted and purified using the traditional cetyltrimethylammonium bromide method according to previously published procedures [20]. PCR-free DNA libraries for full-genome sequencing were constructed from the genomic DNA of the selected strains using a TruSeq DNA kit (Illumina) according to the manufacturer’s protocol, with an average insert size of 350 bp for each sample; these were assayed using the Illumina MiSeq or HiSeq 2000 sequencing systems. A base-calling pipeline (version HC1.4/RTA1.12) was used to process raw fluorescence images and to call sequences. The average Q20 rate of the original sequence was 98.0 %. Using FASTP software (v0.20.0) to filter raw data (parameter: FastP-Q20-L50-R-F5-F5), the average length of clean data was 142 bp and the Q20 rate was 98.9 %. High-quality paired-end reads were mapped to the reference H37Rv genome (GenBank NC_000962.3) using BWA MEM v0.7.1231 [21] or Geneious 6.0 (Biomatters). An average of 520 Mb of data for each sample was generated, representing 230× average sequencing depth and 99.5 % sequence coverage per genome (Table S2). Nucleotide variants including SNPs and short insertions or deletions were called by Snippy (v4.6.0) with default parameters (https://github.com/tseemann/snippy). The whole genome SNP alignment, masking repetitive regions (including PE-PPE, PE-PGRS and transposon sequences, etc.), was used for phylogenetic analysis by IQ-Tree (http://www.iqtree.org). Genome-wide association study (GWAS) analysis of clinical Mtb isolates was performed using Pyseer (v1.3.9). The inputs include a VCF file that contained genomic variants (including SNPs and short indels) of all clinical samples, a text file recording the resistance phenotypes of all isolates and a pairwise distance matrix calculated by the embedded script phylogeny_distance.py based on the maximum likelihood phylogeny. Pyseer was run with the seer model, using multidimensional scaling (MDS) components as fixed effects to control for the population structure. Before running the main program, an MDS was performed based on the matrix to determine the number of dimensions to retain. Both site and burden testing were run, with an additional region file that was extracted from the genomic annotation file as input for the burden test. The significance (*P*-value of the likelihood ratio test) thresholds were determined using the number of unique patterns (one of the output files) by an embedded script (count_patterns.py).

Evolutionary analysis of drug resistance in lab-induced strains was performed as follows. (1) Background removal: the BCF tool was used to merge the mutation list (Vcf file) of the first strain into the three groups of samples, and the mutation sites in all three samples were extracted as background sites. Bedtools were then used to remove the mutations at these sites from the original mutation list of the remaining strains, and the mutation list with the background removed was obtained (2). For each drug-related strain, the sites with mutation frequency ≥1 % and mutation sequence number (read number) ≥5 were extracted from the list of mutations with background removal, and the mutation sites of all strains were combined, and freebayes (parameters: -p2-P --min-coverage 10 --min-repeat-entropy 1.0 -q13-m60 --strict-vcf --report-monomorphic-C5-F 0.05) was used to extract genotype, sequencing depth, number of wild-type sequences and number of mutant sequences of each sample at each site from BAM files.

### Total RNA isolation and RNA-seq analysis

Each exponentially growing Mtb strain (50 ml) was harvested via centrifugation. Total RNA was isolated from three biological replicates of each sample and purified according to previously published methods [22]. After treatment with DNase I, the quality and quantity of each RNA sample were assessed using an Agilent 2100 Bioanalyzer (Agilent Technologies). For RNA-seq, cDNA libraries were prepared using a NEBNext Ultra RNA Library Prep Kit (New England Biolabs) after rRNA removal, and the quality was checked using the Agilent Bioanalyzer 2100 system. Sequencing was performed using the Illumina Novaseq 6000 platform. Reads were mapped against the H37Rv reference genome using Bowtie2 v2.2.6 with default parameters to produce BAM files. Gene expression values were analysed using Fragments per Kilobase Million(FPKM) with HTSeq v0.6.0. Differentially expressed genes (DEGs) were determined using DESeq v1.22.1. Genes with a multiple hypothesis *P*-value adjusted with Benjamini-Hochberg correction below 0.05 (adjusted *P*<0.05) were considered DEGs. Functional enrichment analysis was performed using GOseq v1.22, based on the Gene Ontology (GO) database.

### Real-time quantitative PCR (RT-qPCR)

Total RNA from Mtb was converted to cDNA using a commercial kit [HiScript III first-strand cDNA Synthesis Kit (+gDNA wiper); Vazyme], and qPCR of each sample was performed using ChamQ Universal SYBR qPCR Master Mix (Vazyme). The conventional 2^–∆∆Ct^ method was used to determine the relative mRNA expression of candidate genes in drug-resistant Mtb and its parent strain. SYBR Green was used as the fluorescent probe. Mean±sem was calculated from three independent experiments, and significant differences were determined using an unpaired Student’s *t*-test. The primers used in this study are listed in Table S11.

### Growth curve measurement

Mtb cultures were inoculated into LJ medium and incubated at 37 °C with 5 % CO_2_ for 3 weeks. A portion of the colonies was ultrasonically scattered and transferred to Middlebrook 7H9 medium supplemented with 10 % OADC. The cultures were incubated in stasis at 37 °C with 5 % CO_2_ until the OD_600nm_ reached 0.8. The cultures were ultrasonically scattered to avoid clump formation, and a 100 µl aliquot of each strain was inoculated into 10 ml of Middlebrook 7H9 medium supplemented with 10 % OADC. The OD_600nm_ of 1 ml aliquots of Mtb cultures were measured at the appropriate time for 45 days. Mean OD_600nm_±sem for each time point of the growth curve was calculated from three independent experiments, and significant differences were determined using n unpaired Student’s *t*-test. Growth rates (generations per hour) in the logarithmic phase of growth were calculated as previously described. The OD_600nm_ for each time point of interest was converted to colony forming units (c.f.u.) using the formula c.f.u.=3.26×10^7^×OD_600nm_+6.89×10^5^ [23].

## RESULTS

### A1401G was the major mutation in aminoglycoside-resistant clinical Mtb isolates in Guangdong province

**Fig. 1.**
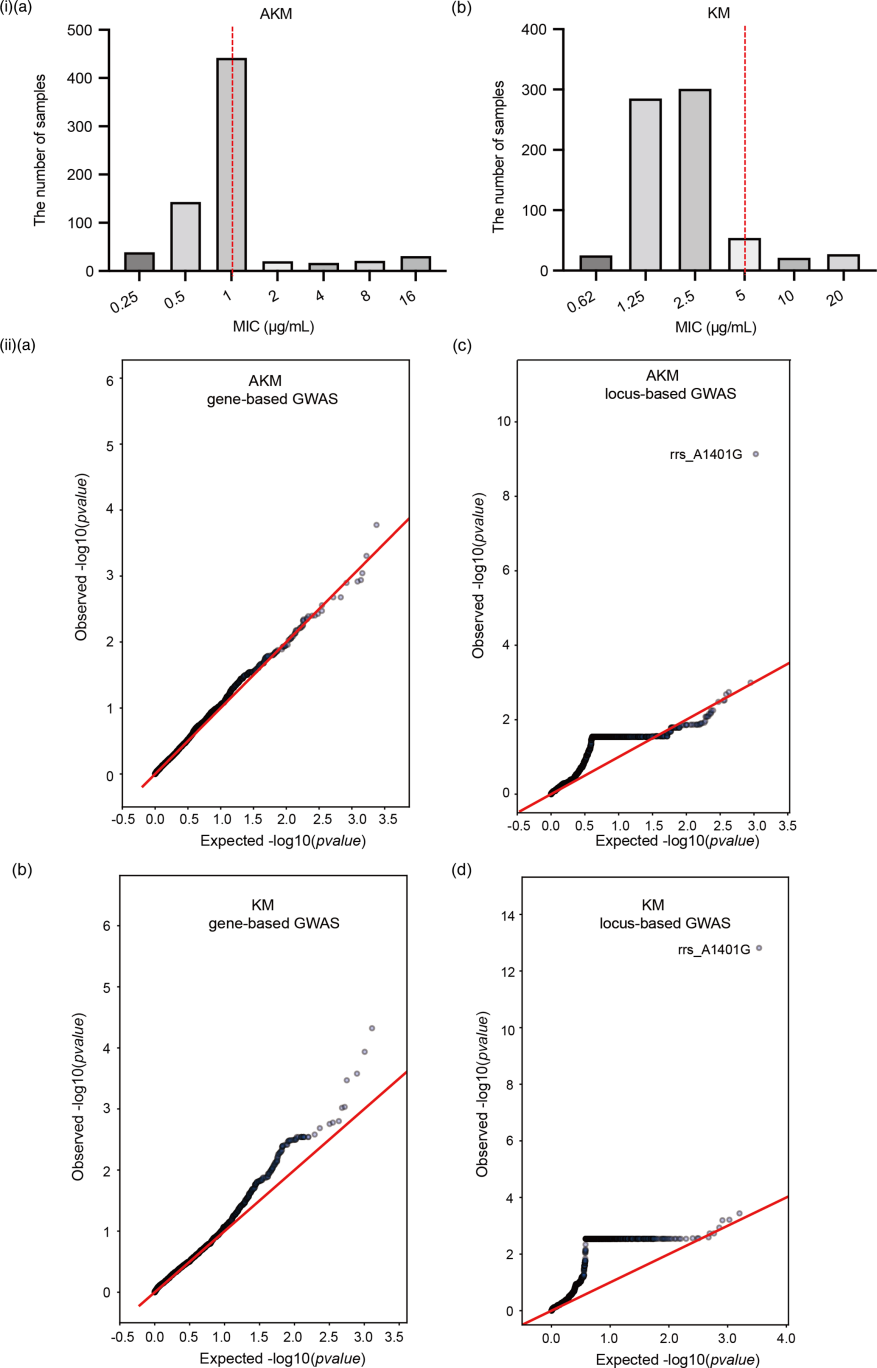
WGS analysis of aminoglycoside-resistant Mtb isolates in Guangdong province. (i) Distributions of the MIC measurements for amikacin (AKM) (**a**) and kanamycin (KM) (**b**) in the GWAS analyses. The red line indicates the WHO recommended critical MIC (CC); measurements to the left of the CC are considered sensitive, and those to the right are considered resistant. (ii) QQ plots for the mutation analyses based on genes (**a,b**) and loci (**c,d**). Comparison of the empirical distribution of *P*-values to the expected distribution under the null hypothesis for AKM (**a,c**) and KM (**b,d**).

Currently, little is known about the mutation profiles of aminoglycoside resistance in clinical Mtb isolates from Guangdong. To investigate the prevalence of aminoglycoside-resistant strains and their specific mutations in drug-resistant Mtb, we screened sensitive and drug-resistant clinical isolates from patients with pulmonary TB at 32 healthcare institutions across the province between 2016 and 2018. A total of 713 eligible isolates were divided into two categories: (1) AKM- and KM-sensitive strains (617 strains); and (2) AKM- or KM-resistant strains (96 strains) (Table S1). All included strains were detected using the broth microdilution method to re-test their drug susceptibility, and whole-genome sequencing (WGS) and bioinformatic analysis were then performed. We first analysed the distribution of classical aminoglycoside resistance-related point mutations in resistant strains and found that *eis* −10G>A, *eis* −14C>T, *rrs* C462T, *rrs* 514A>C, *rrs* A1401G and *tlyA* 513–514 del, among which *rrs* was the most important mutation gene, accounted for 18.9 and 22.8 % in AKM- and KM-resistant strains, respectively (Tables 1 and S2). We assessed genome-wide associations (GWAS) between genes or non-coding region mutations in Mtb and aminoglycoside resistance to determine whether there were detectable interactions between specific resistance sites or genes and the resistance phenotype. GWAS analysis was performed for AKM or KM using a gene/non-coding region binary burden score or a single locus test. The significance thresholds were set at a false discovery rate of <9.44e^−6^ for gene-based GWAS and <4.49e^−5^ for locus-based GWAS. QQ plots of the resultant *P*-value distribution suggested that the correction for population structure was adequate (Fig. 1). This was demonstrated by the adherence of the observed *P*-value distribution to the expected line with the exception of the short tail, indicating the significant loci in Fig. 1ii. Consistent with most of the reported results, *rrs* (A1401G) was the most significant mutation with AKM (likelihood ratio test (lrt)-*P*=7.31e^−10^) and KM (lrt-*P*=1.53e^−13^) (Fig. 1ii and Table S3). Some mutations in genes such as *CysT, Rv1012, rrs* and *guaB2* may be relevant to KM resistance, while *Rv1312* and *gpgS* could be related to AKM resistance (lrt-*P*<5.0e^−04^) (Table S3).

**Table 1.** Classical mutations were identified in AKM- or KM-resistant clinical isolates

Drug	Gene mutations	No. of corresponding strains		Total
AKM	eis_c.-10G>A	2 (2.22 %)	3 (3.33 %)	90
	eis_c.-14C>T	1 (1.11 %)		
	rrs_r.462c>t	4 (4.44 %)	17 (18.89 %)	
	rrs_r.514a>c	3 (3.33 %)		
	rrs_r.1401a>g	10 (11.11 %)		
	tlyA_c.513_514del	1 (1.11 %)	–	
KM	eis_c.-10G>A	1 (1.75 %)	2 (3.51 %)	57
	eis_c.-14C>T	1 (1.75 %)		
	rrs_r.514a>c	3 (5.26 %)	13 (22.81 %)	
	rrs_r.1401a>g	10 (17.54 %)		
	tlyA_c.513_514del	1 (1.75 %)	–	

### Evolution of aminoglycoside resistance to AKM and KM

**Fig. 2.**
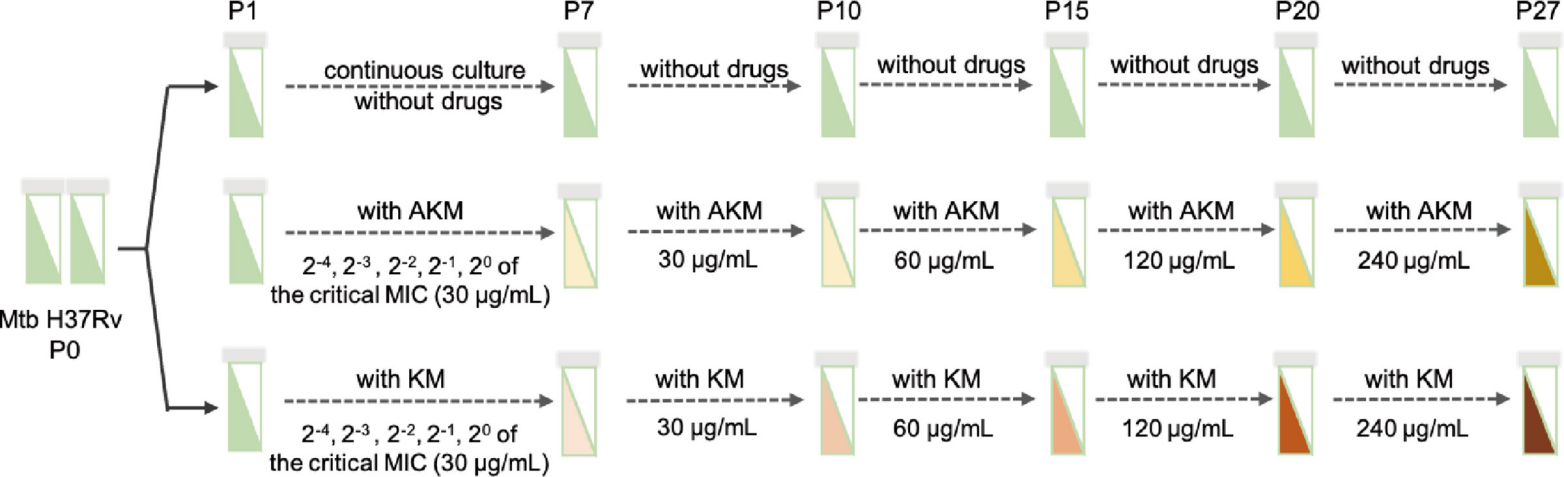
Laboratory evolution of Mtb under aminoglycoside stress conditions. Schematic overview of laboratory evolution of aminoglycoside resistance induced by amikacin (AKM) and kanamycin (KM), respectively. The induced Mtb strains of each generation were preserved and subjected to MIC measurements and other downstream experiments.

To further analyse and verify the relationship between gene mutation and Mtb resistance to aminoglycosides, evolution to drug pressure was carried out on LJ media with increasing concentrations of AKM and KM, and the parent wild-type Mtb H37Rv was cultured in parallel on LJ media without using antibiotics as a control. During the induction process, each generation of positive clones was retained to establish a dynamic model of drug-resistant Mtb, which was helpful in analysing the genetic evolution and drug resistance mechanisms of Mtb strains under drug pressure (Fig. 2). For AKM-induced strains, Mtb showed resistance to AKM in the second generation (P2), but the growth state of the strain was poor, so it was continuously cultivated in LJ media containing escalating doses of AKM to the seventh generation (P7) until the colony morphology was similar to that of the drug-free control group. Then, the cells were cultivated for three more generations to stabilize drug resistance, and thus to obtain an AKM-resistant Mtb strain with a critical MIC. Next, to further explore the mechanism by which Mtb acquires high levels of AKM resistance, we continued to give strain P10 high-level AKM induction culture, and obtained the corresponding AKM-resistant strains (P15, P20 and P26) (Fig. 2), respectively. Similarly, susceptible Mtb evolved over 26 generations until the KM concentration reached 8-fold of the WHO-recommended drug CC (Fig. 2).

The antimicrobial susceptibility profiles of each generation of strain were established by evaluating the MICs of 14 anti-TB drugs using the micro-broth dilution method (Tables S4 and S5). The results showed that when induced by AKM or KM, the strain developed high-level resistance to both AKM (>16-fold) and KM (>4-fold) at a relatively early stage, such as AKM at the second generation and KM at the third generation, and all induced aminoglycoside-resistant strains showed resistance to CAP (Tables S4 and S5). Surprisingly, some AKM-induced resistant Mtb strains were resistant to isoniazid (INH) and levofloxacin (LFX) (Table S4).

**Fig. 3.**
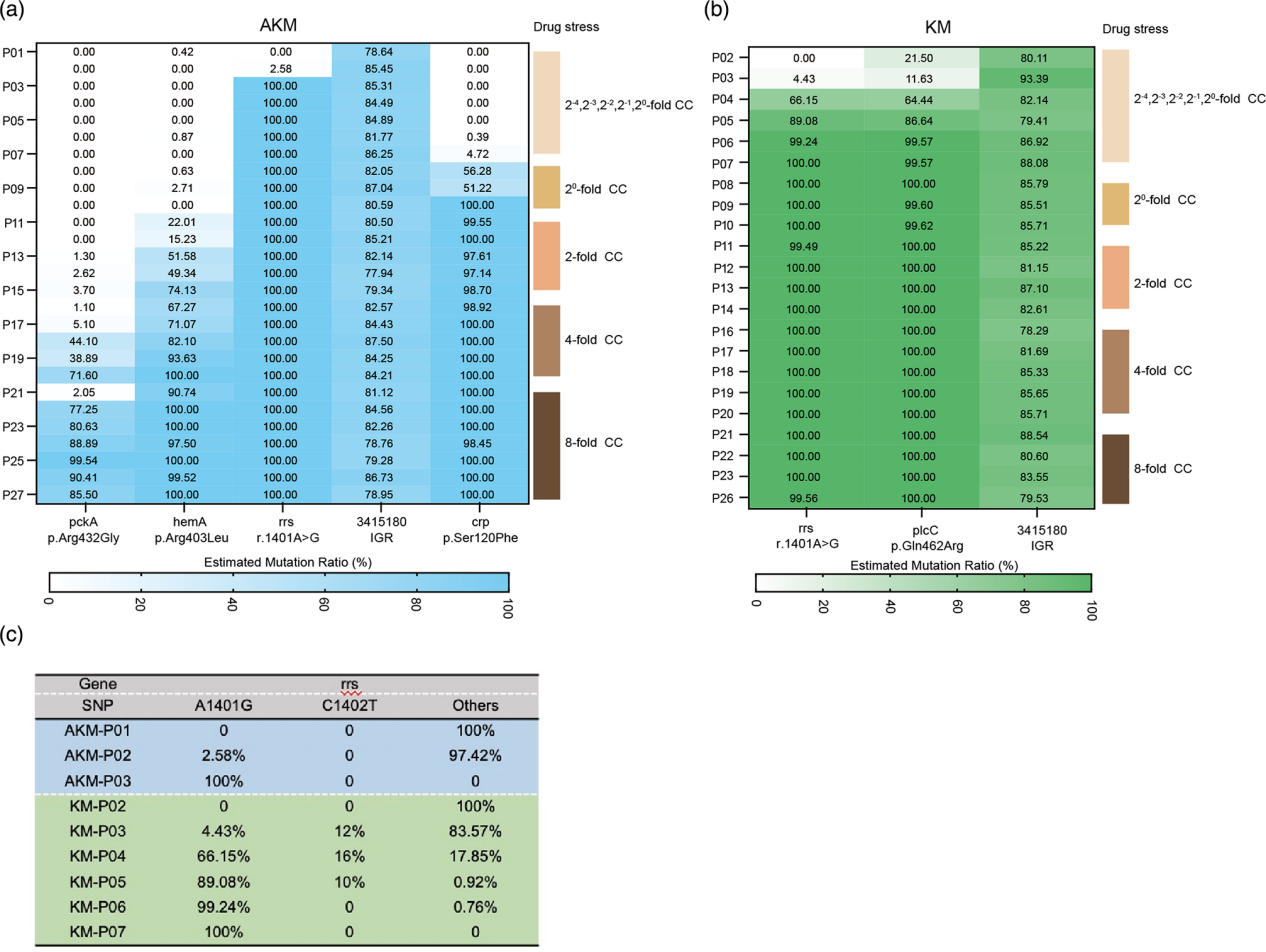
Genetic modifications arising during the evolutionary process related to aminoglycoside resistance. Schematic overview of the genetic modifications that arose during the *in vitro* evolutionary process related to aminoglycoside resistance. WGS and bioinformatic analysis of all strains and their ancestors were performed. With the exclusion of their ancestors’ mutations, the mutations appeared to accumulate gradually with an increase in drug concentration. (**a**) Accumulation of Mtb gene mutations caused by AKM. (**b**) Accumulation of Mtb gene mutations caused by KM. The number under ‘Drug stress’ represents the corresponding drug concentration in LJ medium during induction. CC, the WHO recommended critical concentration (30 mg l^−1^ for AKM and KM). (**c**) In the early stage of induction, the accumulation of *rrs* mutations caused by AKM and KM, respectively.

To identify the genetic modifications that arose during the evolutionary process related to aminoglycoside resistance, WGS and bioinformatic analysis of all strains and their ancestors were performed. With the exclusion of their ancestors’ mutations, we found that the mutations appeared to accumulate gradually with an increase in drug concentration. For AKM-induced Mtb, from the first generation onwards, an intergenic region (IGR) mutation (*nrdH-Rv3054c*) was present, but no significant resistance phenotype was observed (Table S5). From the second to the 10th generation, Mtb acquired high levels of AKM and KM resistance, while the strain accumulated *rrs* (A1401G) and *crp* (Ser120Phe) mutations. Next, from the 11th to the 16th generation, *hemA* (Arg403Leu) was present. From the 17th to the 27th generation, another *pckA* (Arg432Gly) mutation was present (Fig. 3a and Table S4). Compared to AKM-induced strains, KM-induced resistant strains accumulated fewer mutations. The *plcC* mutation (Gln462Arg) and an IGR mutation (*nrdH-Rv3054c*) first appeared in the P2 generation, when the Mtb strains did not develop significant KM resistance. By the fourth generation (P4), another *rrs* gene was mutated, and the strain acquired higher KM and AKM resistance. Subsequently, no new mutations other than those three genes were found to accumulate until the 27th generation (P27) (Fig. 3b and Table S5). We also interesting details of the *rrs* mutations. Under AKM pressure, the *rrs*A1401 mutation appeared in the second generation, only accounting for a small proportion of 2.58 %, and reached 100 % in the third generation. However, under KM pressure, two mutations, *rrs*A1401 (4.43 %) and *rrs*A1402 (12 %), appeared from the third generation. After successive generations, *rrs*A1401 gradually accumulated to 100 % in the seventh generation, while *rrs*A1402 gradually decreased and disappeared in the sixth generation.

We also streaked the P3 strain induced by AKM (AP3) and the P4 strain induced by KM (KP4), randomly selected 50 single clones, and analysed *rrs* by PCR sequencing (Table S6). Four representative clones, AP3-1 (*rrs* A1401G), KP4-8 (*rrs* A1401G), KP4-54 (*rrs* C1402T) and KP4-58 (wild-type *rrs*), were selected and the WGS results showed that among the strains with *rrs* mutations, KP4-8 and AP3-1 had almost identical genetic mutations. KP4-58 had a major mutation in the 5′ UTR of *whiB7* (C3568734A) (Table 2). By testing the MICs of these four strains, we found that AP3-1 and KP4-8 had higher AKM and KM resistance than KP4-54 and KP4-58 (Table S8).

**Table 2.** The characteristics of genetic mutations found in the four representative strains (AP3-1, KP4-8, KP4-54 and KP4-58) that underwent RNA-seq analysis Position, the position in the reference Mtb genome (GenBank NC_000962.3). c., A coding DNA reference sequence; p., a protein reference sequence; r., an RNA reference sequence (transcript). nrdH-Rv3054c indicates the intergene region between nrdH and Rv3054c.

Strain	Position	Mutation	Gene	Product
AP3-1	1 473 246	rrs r.1401A>G	rrs	16S rRNA
	3 415 180	nrdH -Rv3054c CACCTAGGGGGTGG>A	Rv3053c	
KP4-8	1 473 246	rrs r.1401A>G	rrs	16S rRNA
	2 627 314	Missense_variant c.1385A>G p.Gln462Arg	plcC	Phospholipase C
	3 415 180	nrdH -Rv3054c CACCTAGGGGGTGG>A	nrdH -Rv3054c	
KP4-54	1 473 247	rrs r.1402C>T	rrs	16S rRNA
KP4-58	3 568 734	uvrD2-whiB7 C>A	uvrD2-whiB7	

### Transcriptional profiles of lab-induced aminoglycoside-resistant strains

**Fig. 4.**
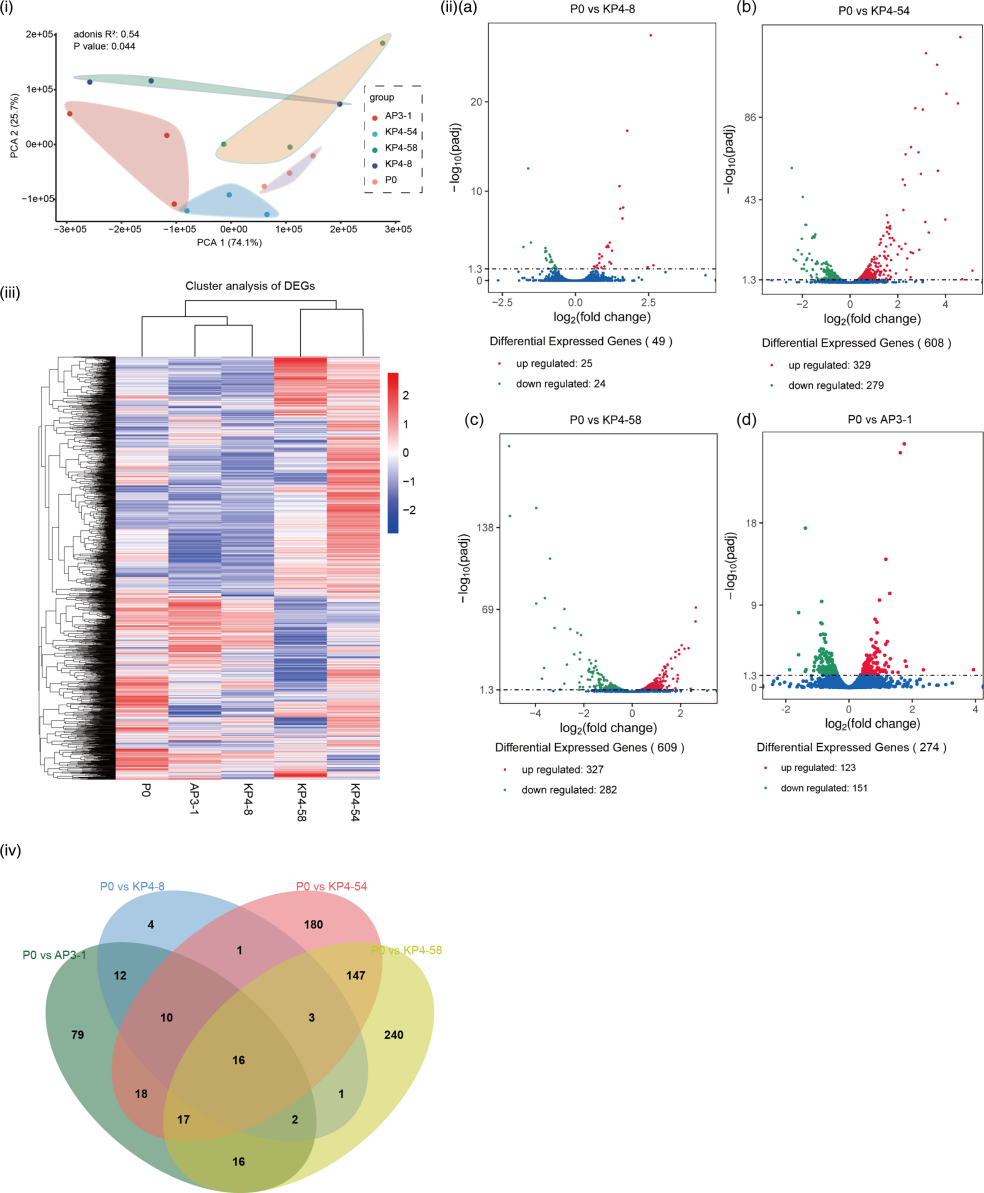
Analysis of the differentially expressed genes (DEGs) of four representative aminoglycoside-resistant Mtb strains. (i) PCA based on DEGs from the four representative aminoglycoside-resistant Mtb strains. *P≤0.05. (ii) Volcano plots displaying DEGs between strains P0 and KP4-8 (**a**); DEGs between P0 and KP4-54 (**b**); DEGs between P0 and KP4-58 (**c**); and DEGs between P0 and AP3-1 (**d**). (iii) Heatmap of the clustered DEGs in (ii). (iv) Venn diagram showing the numbers of overlapping DEGs of the four aminoglycoside-resistant strains relative to the P0 strain.

We performed RNA-seq at P0 of the aforementioned four representative aminoglycoside-resistant Mtb strains, to further investigate the process and mechanism of drug resistance in Mtb. We used principal component analysis (PCA) to compare the gene changes among the AP3-1, KP4-8, KP4-54, KP4-58 and P0 strains (Fig. 4i) . The results showed a significantly separated clustering of genes in drug-resistant Mtb from those of their parent P0 strain, suggesting a change in the overall expression profiles (*P*=0.044). Genes with a multiple hypothesis-adjusted *P*-value below 0.05 (adjusted *P*<0.05) were considered as DEGs (Table S9). Compared to the P0 strain, there were 274 DEGs, including 151 downregulated and 123 upregulated genes, in strain AP3-1; 49 DEGs, including 24 downregulated and 25 upregulated genes, in strain KP4-8; 608 DEGs, including 329 downregulated and 279 upregulated genes, in strain KP4-54; and 609 DEGs, including 327 downregulated and 282 upregulated genes, in strain KP4-58 (Fig. 4ii), and Table S9). These four sets of DEGs were compared using the Venn diagram tool [24], and 16 overlapping genes were identified (Fig. 4iv). Interestingly, consistent with the genome results, there was no significant difference in the transcription profiles of the AP3-1 and KP4-8 strains.

GO analysis of upregulated DEGs in AP3-1, KP4-8, KP4-54 and KP4-58 by DAVID 6.8 (https://david.ncifcrf.gov/tools.jsp) indicated that two biological process terms (response to hypoxia and arginine biosynthetic process), two molecular function terms [quinone-binding NADH dehydrogenase (ubiquinone) activity], and three cellular component terms [NADH dehydrogenase (ubiquinone) activity], plasma membrane respiratory chain complex I, and cell wall and proton-transporting ATP synthase complex, catalytic core F1 were significantly enriched in the KP4-54 strain [false discovery rate (FDR)<0.05]. Arginine and histidine biosynthetic processes were significantly enriched in the KP4-58 strain (FDR<0.05). No GO terms were significantly enriched in either the AP3-1 or KP4-8 strain. Moreover, genes that were significantly changed in AP3-1 and KP4-8 were also changed in KP4-54, but the changes were larger (Table S10).

**Fig. 5.**
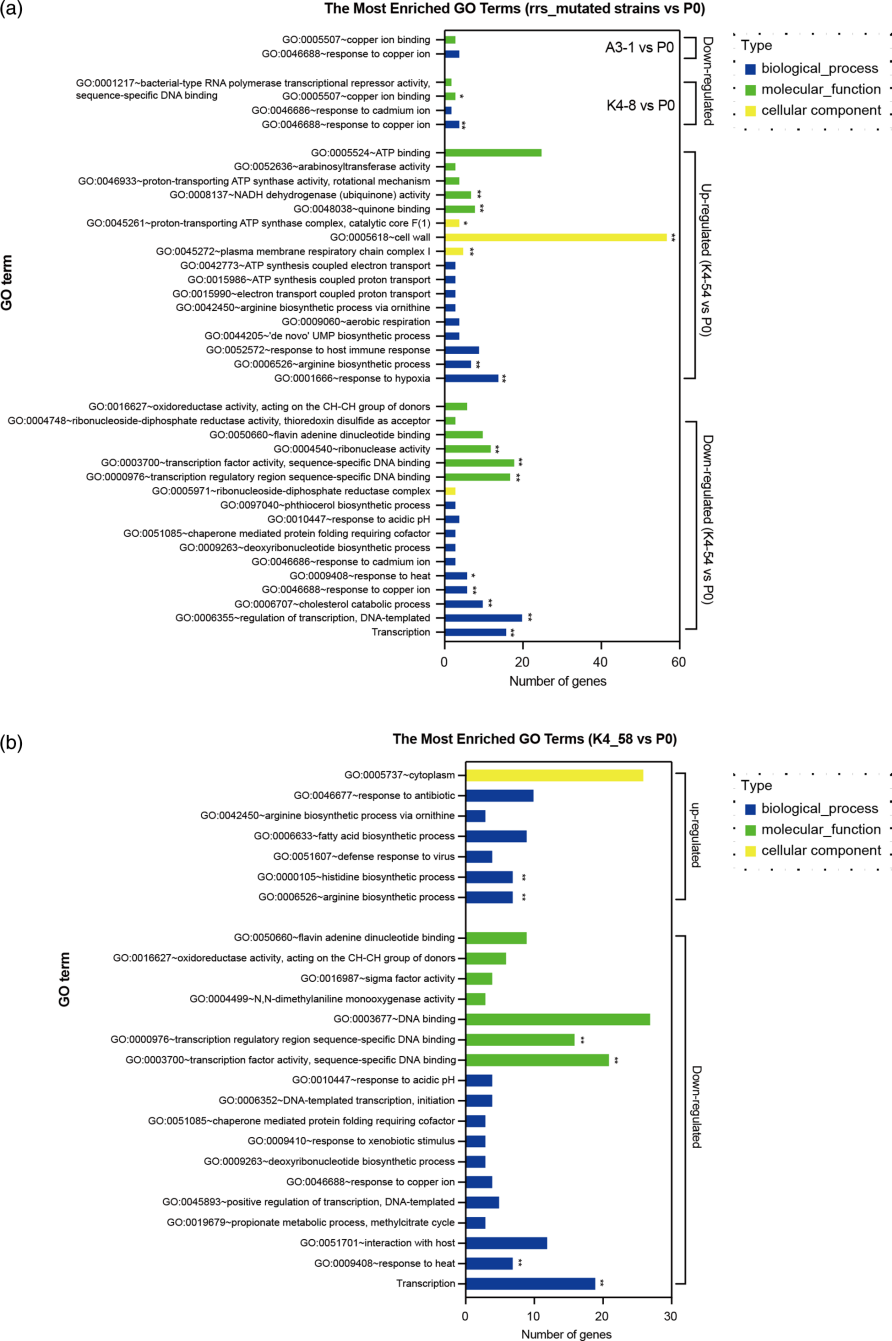
Gene ontology (GO) analysis of differentially expressed genes (DEGs) in four representative aminoglycoside-resistant Mtb strains by DAVID 6.8. (**a**) GO analysis of significant DEGs in strains AP3-1, KP4-8 and KP4-54, respectively, compared to those in their parent strain P0. (**b**) GO analysis of significant DEGs in strain KP4-58, compared with those in strain P0.

**Fig. 6.**
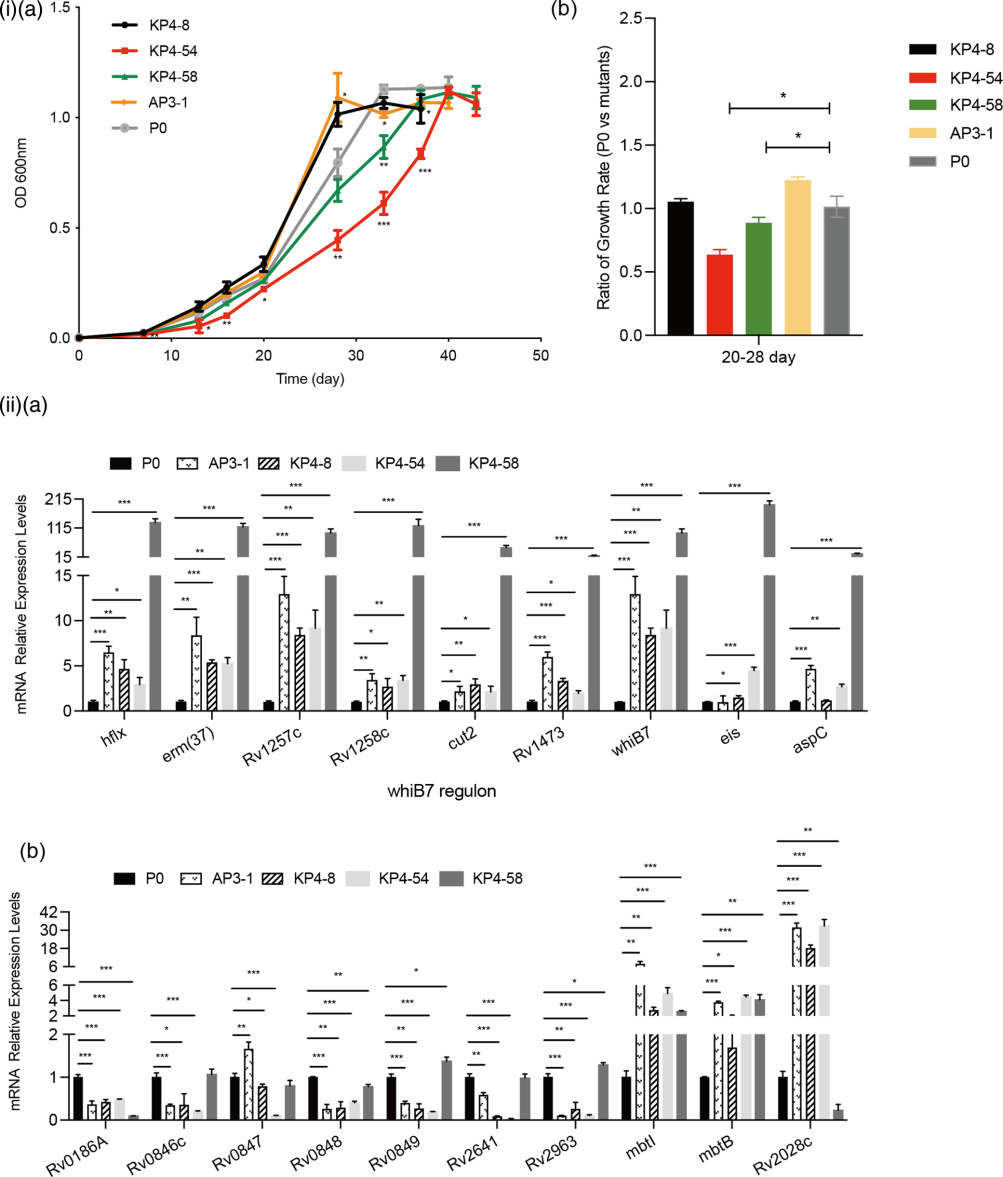
Characterization of representative aminoglycoside-resistant Mtb strains used in this study. (i) Growth of *M. tuberculosis* P0, AP3-1, KP4-8, KP4-54 and KP4-58 strains in 7H9+ADC liquid media for 43 days. (**a**) Growth curve. Mean±sem OD_600nm_ for each time point of the growth curve was calculated from three independent experiments. Compared with P0, significant differences of OD_600nm_ for each time point were determined using an unpaired Student’s *t*-test (*P<0.05,**P<0.01, ***P<0.001). (**b**) The difference in growth rate of each drug-resistant strain in logarithmic phase compared with the parent strain. Generation times in the log-phase of growth on days 20 and 28 were determined. Results are shown as the ratio of mean growth rate (**p0**) ±sem/mean growth rate (drug-resistant strain) ±sem. Statistical significance was determined using a Student’s *t*-test (*P<0.05). (i) Semi-quantitative RT-PCR was used to examine the expression of DEGs of interest screened by RNA-seq. (**a**) The *whiB7* regulon, and (**b**) DEGs involved in response to divalent metal ions. SigA served as the loading control. Mean±sem was calculated from three independent experiments, and significant differences were determined using an unpaired Student’s *t*-test (*P<0.05,**P<0.01, ***P<0.001).

For the GO analysis of downregulated DEGs, we found that transcription, response to heat, sterol metabolism, steroid metabolism and cholesterol metabolism were enriched in both the KP4-54 and KP4-58 strains (FDR<0.05), suggesting an effect on the physiological status of the strain (Fig. 5 and Table S10). In addition, a response to copper ions (GO:0046688) was detected in the three *rrs* mutant strains (Fig. 5a). By measuring the growth curve of the strains, we found that the *rrs*A1401G mutants (KP4-8 and AP3-1) reached the plateau faster than the parental strain (P0), while the *rrs*C1402T mutant (KP4-54) was the latest (Fig. 6i). Growth rates (generations per hour) of these strains were then compared during the logarithmic phase of bacterial growth. As expected, KP4-54 and KP4-58 had significantly lower growth rates than P0 (*P*<0.05), which may be related to the significant downregulation of transcription-related genes in these bacteria.

We then performed Kyoto Encyclopedia of Genes and Genomes-based pathway analysis on the subsets of these DEGs (Table S10). Compared to the P0 strain, upregulated genes in the KP4-54 strain were significantly enriched in oxidative phosphorylation (FDR=5.85e-06) (Table S10). Biosynthesis of siderophore group non-ribosomal peptides was significantly enriched in the A3-1 and K4-8 strains (FDR<0.05). In the KP4-58 strain, upregulated genes were also enriched in the biosynthesis of amino acids, arginine biosynthesis and histidine metabolism (Table S10).

### RT-qPCR validation of differential genes associated with aminoglycoside resistance in Mtb

As mentioned above, there were two IGR mutations in the lab-induced strains, which may be related to aminoglycoside resistance. Numerous studies have shown that IGR mutations may affect the expression levels of flanking genes. Using transcriptome analysis, we found that the expression of *whiB7* was significantly upregulated, by 12.4-fold, in the KP4-58 strain (*P*<0.05), while the expression of flanking genes in the other IGR mutations (*nrdH-Rv3054c*) was not significantly changed in any strain (Table S9). Reeves *et al.* [25] reported that the 5′-UTR of the transcriptional activator *whiB7* could lead to a 23- to 145-fold increase in *whiB7* transcripts and subsequent increased expression of both *eis* (Rv2416c) and *tap* (Rv1258c). We also found that *eis* and Rv1258c were upregulated by 33.7- and 32.9-fold in the KP4-58 strain, respectively. Except for *eis* and Rv1258c, there were seven target genes reported as *whiB7*-dependent transcripts: Rv2415c, Rv1473, *erm* (37), cut2, hflX, aspC and Rv1257c. Unsurprisingly, in our study, these genes were upregulated by 4- to 16-fold in the KP4-58 strain. It is of note that although the *whiB7* regulon accounted for a small proportion (9/282) in all the upregulated differential genes of KP4-58, except Rv1473, the rest ranked among the top eight in the most upregulated genes (Table S9). Conversely, we did not find significant upregulation of the *whiB7* regulon in DEGs of *rrs* mutants.

qPCR was used to independently confirm the nine DEGs within the *whiB7* regulon of the four representative aminoglycoside-resistant Mtb strains. Expression of all these genes in the KP4-58 strain was significantly upregulated compared to both P0 and the other three resistant strains (*P*<0.01) (Fig. 6(iia). In contrast to the RNA-seq results, we found several *whiB7* regulons were also upregulated in the *rrs* mutants, but the magnitude of upregulation was much lower than that of the KP4-58 strain. In addition, qPCR results showed that several DEGs enriched in a response to copper ions (GO:0046688) of *rrs* mutants, such as *Rv0186A, Rv0846c* and *Rv0848*, were significantly downregulated, consistent with the results of RNA-seq (Fig. 6ii).

## DISCUSSION

Aminoglycosides have been beneficial in chemotherapy regimens for MDR-TB over the past few decades, although their use has been limited by systemic administration (muscle or venous route), toxicity and poor patient compliance. Investigation of the aminoglycoside resistance profile of local Mtb isolates and in-depth study of their resistance mechanisms are helpful to guide clinical drug use and better treatment of drug-resistant TB. In this study, we developed a laboratory evolutionary model of aminoglycoside-resistant Mtb *in vitro* using increasing concentrations of AKM and KM. Using WGS analysis, we identified some mutations that are directly associated with aminoglycoside antibiotics in lab-induced Mtb and clinical isolates. Using RNA-seq, this study provides global insight into the characteristics of the transcriptome in four representative induced strains.

### The *rrs* A1401G mutation is the predominant mutation in aminoglycoside-resistant Mtb in Guangdong

The resistance mechanisms of AKM and KM have been considered to be mainly related to gene mutations, including *rrs*, the *eis* promoter and *whiB7* 5′ UTR mutations [12, 26, 27]. In this study, we screened 617 aminoglycoside-sensitive and 96 aminoglycoside-resistant clinical isolates from Guangdong Province between 2016 and 2018 (Table S1). According to the WHO catalogue of resistance, AMK resistance predicted by known genetic mutations is generally much higher, at well over 80 % globally, with *rrs* A1401G alone explaining 73 %. Unexpectedly, only ~20 % *rrs* mutations and 3 % *eis* promoter mutations were found in AKM- and/or KM-resistant strains (Table 1). GWAS analysis for AKM and KM indicated that, in accordance with most of the reported results [13, 17], *rrs* (A1401G) was the most significant locus of resistance to the two drugs. In contrast, mutations in the *whiB7* 5′ UTR were not significantly enriched. In addition, some mutations in genes such as *CysT, Rv1012* and *guaB2* may be relevant to KM resistance, while *Rv1312* and *gpgS* could be related to AKM resistance (lrt-*P* <5.0e-04) (Table S3). Whether these novel genes are related to resistance requires further investigation.

TB is a long-term, intractable and chronic infectious disease. Due to the long treatment period, complex internal environment of the human body, and different sources of clinical strains, it is difficult to eliminate the influence of background factors when comparing and analysing drug resistance-related genotypes. A study from Hebei Province, China, found that 28.6 % (18/63), 17.5 % (11/63), and 12.7 % (8/63) of resistant isolates with an A1401G mutation in *rrs* were resistant to KAN, AMK and CAP, respectively [28]. This is similar to our results, suggesting regional differences in AKM-resistant mutations. Here, we confirmed that *rrs* A1401G was directly related to AKM and KM resistance by constructing an *in vitro* aminoglycoside-resistant Mtb model. By randomly selecting single clones from AKM (AP3) and P4 strains induced by KM (KP4) and testing their *rrs* mutations, we found that 100 % of clones from the AP3 strain had the *rrs* A1401G mutation, while the KP4 strain contained three types of *rrs* genes, i.e. A1401G, C1402T and unmutated *rrs* (Table S8). Similarly, Anna Engstrom *et al.* reported that all spontaneous mutants induced by AKM and 37.5 % (3/8) of mutants selected with KM had an *rrs* A1401G mutant [29]. Although the *rrs* C1402T mutation is cited as being strongly and commonly associated with AKM or KM resistance, Georghiou *et al.* reported that of over 400 injectable drug-resistant strains evaluated, only four had C1402T mutations; thus, this was a poor marker of resistance as it occurred as frequently in drug-sensitive strains as in resistant strains [25, 26]. The representative *rrs* unmutated KP4-58 strain had a major mutation in the 5′-UTR of *whiB7* (C3568734A). Consistent with published results [25, 30], *whiB7* 5′-UTR mutations can cause low levels of KM and CAP resistance and are less frequently observed in clinical isolates. The *rrs* C1402T mutation can cause low levels of AKM and KM resistance and high CAP resistance. The *rrs* A1401G mutation caused high levels of AKM and KM resistance and intermediate CAP resistance (Table S8).

With the continuous multiplication of Mtb and increase in drug concentration, more gene mutations appeared in AKM-induced strains. By the 27th generation, which grew well in LJ medium with an 8-fold CC of AKM, the main mutations were *rrs* (A1401G), *crp* (Ser120Phe), *hemA* (Arg403Leu), *pckA* (Arg432Gly) and an IGR mutation (*nrdH-Rv3054c*) (Fig. 3 and Table S4). Compared to AKM-induced strains, KM-induced resistant strains accumulated fewer mutations from the sixth to 27th generations, including *rrs* A1401G, *plcC* (Gln462Arg) and the same IGR mutations. This suggests that even very similar drugs may have slightly different mechanisms for triggering resistance. For example, drug-resistant strains induced by a high concentration of AKM can simultaneously be resistant to INH, LFX, etc., while KM will not cause cross-resistance to these drugs. The *rrs*1402 mutant and *whiB7* promoter mutant that appeared in the early stage of the experiment gradually disappeared with continuous passage and with increased KM pressure (Fig. 3 and Table S4). We speculate that it may be caused by the following two means: (a) the mutation affects the physiological state of the strain, mainly reflected in the significantly slower growth of the *rrs*1402 and *whiB7* promoter mutants; and (b) the resistance level to KM is significantly lower in *rrs*1401 mutants. Therefore, these two types of mutants were not as advantageous as the *rrs*1401 mutant during the evolution of resistant strains and were eliminated. In addition, we found three clinical strains had an IGR mutation (*nrdH-Rv3054c*) and one *plcC* (Gln462Arg). Except for these mutants, we did not find any new mutated genes identified in the clinical strains. In 2016–2018, given its high toxicity and side effects, KM was rarely used for anti-TB treatment in clinical practice, while AKM was used more frequently, which may partly explain why *eis* and *whiB7* mutations were not significantly enriched in aminoglycoside-resistant Mtb clinical strains in Guangdong. Novel mutations found in laboratory-induced strains were not detected in clinical strains, possibly also due to limitations of the sequencing technology used in this study. While common and appropriate for detecting small variations, the short-read sequencing systematically precludes the detection of larger sequence polymorphisms (>50 bp), which can cause resistance, and they have been systematically under-detected due to the predominance of short-read sequencing in GWAS studies. Mtb clinical samples belong to the complex group, and each sample may contain multiple genotypes. In this study, clinical strains were sequenced by Illumina paired-end methodology, and the drug resistance mutation analysis software was tb-profiler (v2.8.14) (parameters: --caller freebayes --calling_params “-p 2 P 0 C 5 -q 10 m 10 --min-coverage 10 K -F 0.01” --min_depth 10 --af 0.01 --reporting_af 0.01). Genotypes with sequencing depth below 10× are filtered out by the system and will not be shown in the mutation list (Vcf file) or used for subsequent GWAS analysis. Therefore, if the proportion of strains with classical mutations in the Mtb complex is too low, its mutation information will not appear in the analysis results.

### The *rrs*-mutated and unmutated aminoglycoside-resistant Mtb strains have different transcriptional profiles

Because mutations acquired through selection are likely to result in transcriptional changes, different transcriptional patterns between drug-sensitive and drug-resistant strains have attracted extensive attention and research in recent years [31]. For example, Tang *et al.* [32] found that the overlapping DEGs of two MDR strains exhibited low transcription of the type VII secretion system in comparison with Mtb H37Rv, resulting in reduced MDR virulence in mice.

We performed RNA-seq on four representative aminoglycoside-resistant Mtb strains and their parent strain P0. No significant differences were found in the transcription profiles of the two *rrs* A1401G mutant strains, and there were few genes with significant changes in their expression levels compared with the parent strain, suggesting that high drug resistance levels of *rrs* A1401G mutant strains are less correlated with transcriptional regulation. Interestingly, unlike the *rrs* A1401G Mtb, the *rrs* C1402T mutant strain showed more DEGs in its transcriptional profile. It has been reported that mutations in the 16S rRNA A-site affect the conformation of the decoding site to disrupt antibiotic–RNA interactions [33]. Mutations at position 1402 resulted in significant destabilization. Nucleotide 1402 of *rrs* is thought to form a hydrogen bond with nucleotide 1483, and *rrs* C1402T weakens the strength of hydrogen bonding. The genes upregulated in the *rrs* C1402T mutant were significantly enriched in oxidative phosphorylation (Table S10).

Many studies have reported that some drug-resistant bacteria cannot obtain sufficient energy from substrate-level phosphorylation and require oxidative phosphorylation to produce enough ATP for growth [34–36]. During oxidative phosphorylation, the NADH dehydrogenase feeds electrons from NADH into the electron transport chain, leading to a reduction in the menaquinone pool (MK/MKH2). Electrons can be transferred from the menaquinone pool to the cytochrome bc1 complex, which forms a supercomplex with the cytochrome aa3-type terminal oxidase to transfer electrons to oxygen. Alternatively, cytochrome bd-type terminal oxidase receives electrons directly from the menaquinone pool to reduce oxygen. During electron transport along the respiratory chain, protons are pumped across the membrane, resulting in a proton motive force (PMF). The energy of PMF can be used by ATP synthase for ATP synthesis. In the current study, we found that in KP4-54 strains, nine proton-pumping type I NADH dehydrogenases, four cytochrome bd oxidases, three hydrogenlyases and five ATP synthases were upregulated >1.5-fold. Bacteria must maintain a sufficiently high PMF and ATP levels for drug extrusion. However, in the KP4-54 strain, the expression of a large number of efflux pumps was downregulated, for example Rv0849 (9.0-fold), mmpS5 (6.7-fold), mmpL5 (4.7-fold), Rv0191 (2.7-fold) and efpA (1.8-fold) (Fig. 6b and Table S9). These findings suggest that the KP4-54 strain had to gain ATP supplemented by upregulation of oxidative phosphorylation to provide enough energy to maintain the strain’s survival, but these bacterial energy reserves are insufficient to effectively activate the efflux pumps to expel drugs. A study in mutant *Escherichia coli* showed downregulation of oxidative phosphorylation and upregulation of siderophore metabolism genes could reduce the production of reactive oxygen species (ROS) to alleviate the lethal effects of KM, resulting in drug resistance [37]. Conversely, upregulation of oxidative phosphorlation and downregulation of siderophore metabolism genes were observed in induced KM-resistant Mtb strains, suggesting that more ROS may be produced. Therefore, considering the significantly slower growth rate of the KP4-54 strain, we speculated that under drug stress, although KP4-54 could produce enough ATP for survival by upregulating oxidative phosphorylation and downregulating siderophore metabolism, it also produced excessive ROS, causing damage to the strain and leaving it in a poor growth state. In the process of evolution, such weak strains would be gradually eliminated.

In the *rrs* unmutated strain (KP4-58), the bacteria adopted an almost completely different regulatory strategy from that of the *rrs* mutant strain to adapt to antibiotic pressure, in which *whiB7* is a key factor. Morris *et al.* demonstrated that *whiB7* can affect drug resistance by activating the expression of regulons, including genes involved in ribosomal protection and antibiotic efflux [38]. In Mtb KP4-58, using RNA-seq and qPCR assays, *whiB7* and its several target genes, such as *eis*, *tap* (Rv1258c), *erm* [37] and *Rv1473*, showed an increase of between 15- and 210-fold, respectively (Fig. 6b and Table S9). Many studies have indicated that increased expression of *eis* or *tap* (*Rv1258c*) can confer aminoglycoside resistance to Mtb. Methyltransferase *erm* [37] slips on rRNA, and an increase in its transcripts can cause intrinsic resistance to clarithromycin and ketolide HMR3004 in Mtb [39]. *Rv1473* is a novel ATP-dependent cassette efflux pump that participates in the resistance of macrolides through an efflux mechanism [40]. In Mtb KP4-58, *whiB7* 5’-UTR mutations enhance *whiB7* expression and activate drug-resistant target genes regulated by *whiB7*, resulting in drug resistance. Note that this resistance is mainly based on transcriptional regulation, and the aminoglycoside resistance level of the strain is relatively low and close to the critical resistance MIC value. Under drug pressure, resistance was lost as the strain evolved.

GO and KEGG analysis of downregulated DEGs indicated that the ‘response to copper ion’ and ‘siderophore group non-ribosomal peptides’ pathways were significantly enriched in *rrs* A1401G mutants, while cholesterol metabolism was significantly enriched in KP54 and KP58 (FDR<0.05). Divalent metal ions are very important for Mtb to survive. ‘Siderophore group non-ribosomal peptides’ has environment-specific importance in the host, as it is used for iron sequestration/ultilization, which is tightly controlled during infection [41, 42]. When infected by Mtb, the host would starve the invader by reducing the concentration of iron, copper, etc., to a minimum [43]. The host-derived fatty acids are important carbon sources during Mtb infection, and inhibiting host lipid intake can affect the pathogenicity of Mtb [44]. Recent studies have revealed the metabolic changes of fatty acid/cholesterol-induced dormancy state of Mtb and its relationship with the acquisition of drug resistance, suggesting that fatty acid/cholesterol-induced dormancy state remodelled the central carbon metabolism of Mtb and was functionally related to the acquisition of drug resistance of Mtb [45, 46]. Jakub Pawełczyk *et al.* [47] studied the transcription profile of Mtb with cholesterol as the only carbon source, and found that many changes in the transcriptome of Mtb that were previously thought to be caused by a lipid-rich environment were actually induced by cholesterol. They suggested that cholesterol helps Mtb enter a drug-tolerance state early; the key role of cholesterol in *Mycobacterium* metabolism is not only to provide carbon and energy, but also to involve transcriptome remodelling programmes that contribute to the development of tolerance to an adverse host cell environment prior to the occurrence of specific stress-induced phagosomal signalling.

This study has some deficiencies in the comparative transcriptomic analysis. Optimal comparative analysis should use the drug-free evolved strain at P4 as the control. In this study, the parent strain P0 was used instead. Therefore, the transcriptomic difference caused by *in vitro* growth and stochastic drift (epigenetic) cannot be completely ruled out.

In conclusion, we have investigated the mutational profile of aminoglycoside-resistant clinical Mtb isolates in Guangdong Province and found that *rrs* A1401G was the most frequent mutation. Evolutionary models of aminoglycoside resistance were then constructed using AKM and KM *in vitro*. WGS analysis and transcriptional profiling of Mtb strains during evolution revealed that Mtb strains harbouring *rrs* A1401G have an evolutionary advantage over other drug-resistant strains under the pressure of aminoglycosides because of their ultra-high resistance level and low physiological impact on the strain. To some extent, this study explains why *rrs* A1401G had a high frequency in clinical strains resistant to KM or AKM in Guangdong, whereas *eis*, *whiB7*, *tlyA* and other mutations appeared rarely. These results provide a better understanding of the mechanisms involved in the development of aminoglycoside resistance.

## Supplementary Data

Supplementary material 1

Supplementary material 2

Supplementary material 3

Supplementary material 4

Supplementary material 5

Supplementary material 6

## References

[1] WHO (2021). GLOBAL TUBERCULOSIS REPORT.

[2] Chen Y, Wen W, Wu H, Xu L, Peng K (2022). Analysis of monitoring results of tuberculosis drug-resistance in Guangdong Province from 2016 to 2020. Chinese J Antituberculosis.

[3] Bisson GP, Bastos M, Campbell JR, Bang D, Brust JC (2020). Mortality in adults with multidrug-resistant tuberculosis and HIV by antiretroviral therapy and tuberculosis drug use: an individual patient data meta-analysis. Lancet.

[4] Sabur NF, Brar MS, Wu L, Brode SK (2021). Low-dose amikacin in the treatment of multidrug-resistant tuberculosis (MDR-TB). BMC Infect Dis.

[5] (2016). WHO Treatment Guidelines for Drug-Resistant Tuberculosis, 2016 Update. WHO Guidelines Approved by the Guidelines Review Committee.

[6] (2019). WHO consolidated guidelines on drug-resistant tuberculosis treatment. WHO Guidelines Approved by the Guidelines Review Committee.

[7] Ahmad MH, Rechenmacher A, Böck A (1980). Interaction between aminoglycoside uptake and ribosomal resistance mutations. Antimicrob Agents Chemother.

[8] Davis BD, Chen LL, Tai PC (1986). Misread protein creates membrane channels: an essential step in the bactericidal action of aminoglycosides. Proc Natl Acad Sci.

[9] Jian J, Yang X, Yang J, Chen L (2018). Evaluation of the genotype mtbdrplus and mtbdrsl for the detection of drug-resistant *Mycobacterium tuberculosis* on isolates from Beijing, China. Infect Drug Resist.

[10] Programme GT (2019). Rapid Communication- Key changes to the treatment of drug-resistant tuberculosis WHO/CDS/TB/2019.:6.

[11] Brossier F, Pham A, Bernard C, Aubry A, Jarlier V (2017). Molecular investigation of resistance to second-line injectable drugs in multidrug-resistant clinical isolates of *Mycobacterium tuberculosis* in France. Antimicrob Agents Chemother.

[12] Vargas R, Freschi L, Spitaleri A, Tahseen S, Barilar I (2021). Role of epistasis in Amikacin, Kanamycin, Bedaquiline, and Clofazimine resistance in *Mycobacterium tuberculosis* complex. Antimicrob Agents Chemother.

[13] Farhat MR, Freschi L, Calderon R, Ioerger T, Snyder M (2019). GWAS for quantitative resistance phenotypes in *Mycobacterium tuberculosis* reveals resistance genes and regulatory regions. Nat Commun.

[14] Pholwat S, Stroup S, Heysell S, Ogarkov O, Zhdanova S (2016). eis promoter C14G and C15G mutations do not confer kanamycin resistance in *Mycobacterium tuberculosis*. Antimicrob Agents Chemother.

[15] Sowajassatakul A, Prammananan T, Chaiprasert A, Phunpruch S (2018). Overexpression of eis without a mutation in promoter region of amikacin- and kanamycin-resistant *Mycobacterium tuberculosis* clinical strain. Ann Clin Microbiol Antimicrob.

[16] Sowajassatakul A, Prammananan T, Chaiprasert A, Phunpruch S (2014). Molecular characterization of amikacin, kanamycin and capreomycin resistance in M/XDR-TB strains isolated in Thailand. BMC Microbiol.

[17] Coll F, Phelan J, Hill-Cawthorne GA, Nair MB, Mallard K (2018). Genome-wide analysis of multi- and extensively drug-resistant *Mycobacterium tuberculosis*. Nat Genet.

[18] Du Q, Dai G, Long Q, Yu X, Dong L (2013). *Mycobacterium tuberculosis* rrs A1401G mutation correlates with high-level resistance to kanamycin, amikacin, and capreomycin in clinical isolates from mainland China. Diagn Microbiol Infect Dis.

[19] Miryala SK, Anbarasu A, Ramaiah S (2019). Impact of bedaquiline and capreomycin on the gene expression patterns of multidrug-resistant *Mycobacterium tuberculosis* H37Rv strain and understanding the molecular mechanism of antibiotic resistance. J Cell Biochem.

[20] Zhao J, Wei W, Yan H, Zhou Y, Li Z (2019). Assessing capreomycin resistance on tlyA deficient and point mutation (G695A) *Mycobacterium tuberculosis* strains using multi-omics analysis. Int J Med Microbiol.

[21] Li H, Durbin R (2009). Fast and accurate short read alignment with Burrows-Wheeler transform. Bioinformatics.

[22] Wang M, Fleming J, Li Z, Li C, Zhang H (2016). An automated approach for global identification of sRNA-encoding regions in RNA-Seq data from *Mycobacterium tuberculosis*. Acta Biochim Biophys Sin.

[23] Qin Y (2016). Correlation between optical density and colony forming units of *Mycobac-terium tuberculosis* suspension.

[24] Bardou P, Mariette J, Escudié F, Djemiel C, Klopp C (2014). jvenn: an interactive Venn diagram viewer. BMC Bioinformatics.

[25] Reeves AZ, Campbell PJ, Sultana R, Malik S, Murray M (2013). Aminoglycoside cross-resistance in *Mycobacterium tuberculosis* due to mutations in the 5’ untranslated region of whiB7. Antimicrob Agents Chemother.

[26] Georghiou SB, Magana M, Garfein RS, Catanzaro DG, Catanzaro A (2012). Evaluation of genetic mutations associated with *Mycobacterium tuberculosis* resistance to amikacin, kanamycin and capreomycin: a systematic review. PLoS One.

[27] Miotto P, Tessema B, Tagliani E, Chindelevitch L, Starks AM (2017). A standardised method for interpreting the association between mutations and phenotypic drug resistance in *Mycobacterium tuberculosis*. Eur Respir J.

[28] Li Q, Gao H, Zhang Z, Tian Y, Liu T (2019). Mutation and transmission profiles of second-line drug resistance in clinical isolates of drug-resistant *Mycobacterium tuberculosis* from Hebei Province, China. Front Microbiol.

[29] Engström A, Perskvist N, Werngren J, Hoffner SE, Juréen P (2011). Comparison of clinical isolates and in vitro selected mutants reveals that tlyA is not a sensitive genetic marker for capreomycin resistance in *Mycobacterium tuberculosis*. J Antimicrob Chemother.

[30] Reeves AZ, Campbell PJ, Willby MJ, Posey JE (2015). Disparities in capreomycin resistance levels associated with the rrs A1401G mutation in clinical isolates of *Mycobacterium tuberculosis*. Antimicrob Agents Chemother.

[31] de Welzen L, Eldholm V, Maharaj K, Manson AL, Earl AM (2017). Whole-transcriptome and -genome analysis of extensively drug-resistant *Mycobacterium tuberculosis* clinical isolates identifies downregulation of *ethA* as a mechanism of ethionamide resistance. Antimicrob Agents Chemother.

[32] Tang J, Liu Z, Shi Y, Zhan L, Qin C (2020). Whole genome and transcriptome sequencing of two multi-drug resistant *Mycobacterium tuberculosis* strains to facilitate illustrating their virulence *in vivo*. Front Cell Infect Microbiol.

[33] Truitt AR, Choi B-E, Li J, Soto AM (2015). Effect of mutations on the binding of Kanamycin-B to RNA hairpins derived from the *Mycobacterium tuberculosis* ribosomal a-site. Biochemistry.

[34] Kohanski MA, Dwyer DJ, Hayete B, Lawrence CA, Collins JJ (2007). A common mechanism of cellular death induced by bactericidal antibiotics. Cell.

[35] Méhi O, Bogos B, Csörgő B, Pál F, Nyerges A (2014). Perturbation of iron homeostasis promotes the evolution of antibiotic resistance. Mol Biol Evol.

[36] Kohanski MA, DePristo MA, Collins JJ (2010). Sublethal antibiotic treatment leads to multidrug resistance via radical-induced mutagenesis. Mol Cell.

[37] Mogre A, Veetil RT, Seshasayee ASN (2017). Modulation of global transcriptional regulatory networks as a strategy for increasing Kanamycin resistance of the translational elongation factor-G mutants in *Escherichia coli*. G3.

[38] Morris RP, Nguyen L, Gatfield J, Visconti K, Nguyen K (2005). Ancestral antibiotic resistance in *Mycobacterium tuberculosis*. Proc Natl Acad Sci.

[39] Andini N, Nash KA (2006). Intrinsic macrolide resistance of the *Mycobacterium tuberculosis* complex is inducible. Antimicrob Agents Chemother.

[40] Duan W, Li X, Ge Y, Yu Z, Li P (2019). *Mycobacterium tuberculosis* Rv1473 is a novel macrolides ABC efflux pump regulated by WhiB7. Future Microbiol.

[41] Rodriguez GM, Sharma N, Biswas A, Sharma N (2022). The iron response of *Mycobacterium tuberculosis* and its implications for tuberculosis pathogenesis and novel therapeutics. Front Cell Infect Microbiol.

[42] Zhang L, Hendrickson RC, Meikle V, Lefkowitz EJ, Ioerger TR (2020). Comprehensive analysis of iron utilization by *Mycobacterium tuberculosis*. PLoS Pathog.

[43] Arnold FM, Weber MS, Gonda I, Gallenito MJ, Adenau S (2020). The ABC exporter IrtAB imports and reduces mycobacterial siderophores. Nature.

[44] Joshi SM, Pandey AK, Capite N, Fortune SM, Rubin EJ (2006). Characterization of mycobacterial virulence genes through genetic interaction mapping. Proc Natl Acad Sci U S A.

[45] Xu J-Y, Zhao L, Xu Y, Li B, Zhai L (2020). Dynamic Characterization of Protein and Posttranslational Modification Levels in Mycobacterial Cholesterol Catabolism. mSystems.

[46] Aguilar-Ayala DA, Tilleman L, Van Nieuwerburgh F, Deforce D, Palomino JC (2017). The transcriptome of *Mycobacterium tuberculosis* in a lipid-rich dormancy model through RNAseq analysis. Sci Rep.

[47] Pawełczyk J, Brzostek A, Minias A, Płociński P, Rumijowska-Galewicz A (2021). Cholesterol-dependent transcriptome remodeling reveals new insight into the contribution of cholesterol to *Mycobacterium tuberculosis* pathogenesis. Sci Rep.

